# Condom Use among HIV-Positive Postnatal Women in Primary Health Care Facilities in Tshwane Sub-District 1, Gauteng Province, South Africa

**DOI:** 10.3390/ijerph20196877

**Published:** 2023-10-02

**Authors:** Mpho Kgoele, Modikwe Rammopo, Oluwafemi O. Oguntibeju

**Affiliations:** 1 Department of Public Health, School of Health Care Sciences, Sefako Makgatho Health Sciences University, Pretoria 0204, South Africa; mpho.kgoele@smu.ac.za (M.K.); modikwe.rammopo@smu.ac.za (M.R.); 2 Department of Biomedical Sciences, Faculty of Health and Wellness Sciences, Cape Peninsula University of Technology, Bellville 7535, South Africa

**Keywords:** condoms, postnatal, woman, health education, HIV, prevention

## Abstract

Condoms have been and are still an important part of HIV preventative measures worldwide, and many countries have designed programmes that encourage their use. Consistent and correct condom use among HIV-positive individuals is important in preventing multiple infections. Hence, the uptake and determining factors associated with condom use were investigated in this study. This study was aimed at determining the level of, and factors associated with, condom use among HIV-positive postnatal women in primary health care facilities in Tshwane sub-district 1, Gauteng Province, South Africa. A descriptive cross-sectional study was conducted among 326 HIV-positive postnatal women aged between 15 and 50 years who were conveniently selected and voluntarily participated in the study. A self-developed pretested questionnaire was used to collect data on level of condom use and factors associated with its use from the selected participants. Statistical tests of correlation were then used to determine the association between frequency of condom use during sexual encounter and condom use at last sexual encounter with the independent variables. Regular condom use during sexual encounters was reported by 63.2% of the participants while 83% of the participants reported using a condom at their last sexual encounter. Frequency of condom use during sexual encounter was found to be associated with employment status (*p* < 0.05), residence (*p* < 0.001), number of children (*p* < 0.05), first HIV diagnosis (*p* < 0.05) and disclosure of HIV status to partner (*p* < 0.05). Condom use at last sexual encounter was also found to have a significant statistical association with level of education (*p* < 0.05) and the ability to negotiate condom use (*p* < 0.001). A good proportion of the participants used condoms regularly. Interventions to improve condom use among this population should focus on female empowerment by investing in their education, and economic empowerment to improve their economic status, which, in turn, would help the women to better negotiate condom use. The other factors mentioned above should also be considered when developing health education policies and programmes about condom use amongst HIV-positive postnatal women.

## 1. Introduction

The prevention of human immunodeficiency virus (HIV) transmission amongst HIV-positive couples is one of the objectives of public health. It is reported that a common and proven effective method for achieving this objective is through consistent and correct use of condoms amongst couples. When used consistently and correctly, condoms are said to offer 80–95% reduction in transmission of HIV [1]. Condom use offers multiple benefits of preventing unwanted pregnancies as well as the prevention of sexually transmitted infections. This is over and above preventing re-infection of pregnant mothers with HIV, thereby decreasing the chance of mother-to-child transmission of the virus during pregnancy and postnatal period [2]. A European study by Cicconi et al. [3] reported inconsistent and low level of condom use in both HIV-negative and positive women. This was evidenced by an increased number of unplanned pregnancies and sexually transmitted infections amongst this group. This study further showed that it is important to explore factors associated with condom use, as the use of condoms is an essential component of HIV and STI prevention strategies.

HIV is one of the world’s leading communicable diseases with about 35 million people living with HIV worldwide, and Sub-Saharan Africa remains the most affected region [4]. Reports indicate that most of the new infections occur through sexual contact, with females being three times more prone to acquiring HIV from male partners than a male from a female [5]. Hence, women are more vulnerable to HIV and AIDS compared to men [6]. This vulnerability is further increased by biological, social, and economic factors. According to Dube et al. [6], new HIV infections are reported amongst young women and girls. This indicates that more effective preventative strategies are urgently needed to curb new HIV infections in women of childbearing age. The use of highly effective antiretroviral therapy (HAART) has been useful in reducing morbidity and mortality from HIV/AIDS, but treatment has had unintended effects on sexual behaviour apart from beneficial clinical effects [4]. One of these unintended effects includes a low level of consistent condom use. Furthermore, Madiba and Letswalo [7] reported that there is no convincing reduction in risky sexual behaviour among people infected with HIV because of access to antiretroviral therapy. This is supported by the increased prevalence of unprotected sex and incidence of sexually transmitted infections including HIV since HAART became available [4]. HIV prevention strategies have shifted their focus from only targeting HIV-negative people and now include those who are already HIV-positive [8]. One of these prevention strategies is condom use which has always been promoted among HIV-negative people. However, research has shown that even HIV-positive people could greatly benefit from correct and consistent use of condoms [7]. Condom use is considered as one of the foundations for the prevention of HIV transmission and its promotion is internationally and nationally recommended as a dual method for women who are infected with HIV when combined with another effective method of contraception [9,10].

The fact that sexual intercourse is known as the primary form of HIV transmission calls for modification and change in sexual behaviour by promoting condom use as it could make a significant difference in the prevention of sexually transmitted infections including HIV [5,11]. Despite the well-known effectiveness of condoms, they are still not correctly and consistently used especially among individuals infected with HIV [12]. Hence, their use has been widely endorsed as one of the public health strategies against sexually transmitted infections [9].

HIV-positive postnatal women are at risk of transmitting HIV to their babies during breastfeeding. This risk increases further when these women engage in risky sexual behaviours, such as not using condoms during sexual intercourse [13]. Evidence of such behaviour is observed when these women present themselves with sexually transmitted infections at primary health care facilities for consultation. According to the WHO, about 90% of children living with HIV acquired the infection during pregnancy, birth, and breastfeeding [14]. Condoms will not only benefit this population in terms of protecting them from sexually transmitted infections, but they will also act as contraceptives to prevent unplanned pregnancies [2], as it has been shown that in Sub-Saharan Africa the use of any contraceptives by HIV-positive women has resulted in a 30% decrease in HIV mother-to-child transmission [15]. Consequently, the consistent use of condoms in conjunction with a reliable method of contraception could further decrease the transmission of HIV from mother to child; hence, this study is designed to determine the level of, and factors associated with, condom use among HIV-positive postnatal women in primary health care facilities in Tshwane sub-district 1, Gauteng Province, South Africa. The study could help in identifying gaps that need serious attention and facilitate the development of contextualised health programmes to address the identified gaps.

## 2. Methodology

### 2.1. Study Design

This is a descriptive cross-sectional quantitative study which allows examination of the relationship between factors associated with condom use among HIV-positive postnatal women at a given point.

### 2.2. Study Setting

The study took place in primary health care facilities in Tshwane sub-district 1, Gauteng province. The health care facilities were Soshanguve 3 CHC, Kgabo CHC and Boekenhout CHC, offering 24-h health services. In these three community health centres (CHC), about 1500 HIV-positive postnatal women are seen monthly. Tshwane sub-district 1 is located 37 kilometres from Pretoria central. The sub-district 1 consists mostly of semi-rural and township settlements where the most spoken local language is Setswana.

### 2.3. Sampling and Selection of the Sample

The study population was HIV-positive postnatal women in primary health care facilities in Tshwane sub-district 1, Gauteng Province, 6 weeks to a year postnatal, ranging from the ages of 15 to 50 years. Based on current statistics, approximately 1500 HIV-positive postnatal women are seen monthly at the three identified primary health care facilities in Tshwane sub-district 1, Gauteng Province. A convenience sampling technique was used; prospective participants were selected according to their availability when they came for postnatal care in the selected primary health care facilities. The inclusion criteria for this study were all women who were HIV-positive, within 6 weeks to a year in the postnatal period, and were within the ages of 15 to 50 years. Those women who did not fall within these criteria were excluded from the study.

A sample size of 306 HIV-positive postnatal women was determined using a Raosoft calculator with a 5% margin of error and 95% confidence level. The Raosoft calculator (version 377) is a software program that is used to calculate the sample size in quantitative studies. Since determining sample size can be complex, the Raosoft calculator assists in determining the sample size in a simple, reliable, and automated manner to minimise the use of manual calculations. Over-sampling by 20 participants was included to cover the possibility of spoiled questionnaires, resulting in a total sample of 326.

### 2.4. Data Collection Methods and Procedures

#### Recruitment of the Study Participants

Data were collected after ethical approval and clearance was obtained from the Sefako Makgatho Health Sciences University Research Ethics Committee and the Tshwane Research Committee District Health Office, respectively. Appointments were scheduled with the managers of the primary health care facilities for the recruitment of participants. The nursing staff identified those participants who met the inclusion criteria during their immunization appointment. The purpose of the study was explained to the participants and only those who consented to participate in the study were recruited. The same method of recruitment was used until the proposed sample size was reached.

### 2.5. Pilot Testing

A pilot study was conducted on participants to develop and refine the methodology of the study such as identifying and addressing any shortcomings of the research questionnaire. It was conducted at a different 24-h service primary health care facility in the Tshwane sub-district. The results of the pilot study are not included in the main study. The study questionnaire was modified based on the responses of the participants from the pilot study.

### 2.6. Questionnaire

Data were collected from the three identified primary health care facilities with the help of a trained assistant. A self-developed, pretested questionnaire was used to collect data whereby information on demographic data, level of condom use, and factors associated with condom use among HIV-positive postnatal women were gathered. The questionnaire was in English and then translated into the Setswana language as it is the most spoken language in Tshwane. Participants who had verbally consented to participate in the study were taken to a private room where the full details of the study were explained to them and written informed consent obtained. The questionnaire was self-administered. Data were collected from 326 HIV-positive postnatal women who met the inclusion criteria. Participants were assured that their anonymity would be protected by not including their names on the questionnaires. Each questionnaire took about 15–20 min to complete. The total number of questionnaires returned was 326 (100% response rate).

### 2.7. Data Analysis

Raw data were captured on a Microsoft Excel 2007 spreadsheet and imported into STATA software version 13.0 for analysis. Descriptive statistics and frequency distributions were used to analyse and interpret the mean, median and ratio. Bivariate analysis and multivariate logistic regression were used to determine associations between the independent variables and the two dependent variables associated with condom use. The level of condom use was calculated using the proportions of those using condoms and those not using them. Statistical significance was set at *p* < 0.05 with a confidence interval of 95%.

### 2.8. Validity

The data collection tool was pretested in a pilot study, which was conducted amongst 10 participants in a different 24-h health service primary health care facility in Tshwane sub-district 1. The results from the pilot study were used to refine the tool based on the discrepancies identified.

### 2.9. Reliability

Reliability was ensured by using the same data collection tool for all the participants. Data were collected in a private room (one participant at a time) for all the participants to ensure that participants were relaxed and free to participate in the study. Simple language with no jargon or vague questions was used to ensure that the questions were easily understood at the same level amongst all participants. The tool was translated to Setswana for those who were not conversant with the English language. The questions were explained as they appeared on the questionnaire with no additional information that might influence the response of the participants.

### 2.10. Ethical Considerations

Ethical approval was granted by the Sefako Makgatho Health Sciences University Research Ethics Committee (Ref: SMUREC/H/259/2017: PG dated 5 October 2017) and permission was obtained from the Tshwane District Health Office and the selected primary health care facilities’ managers to conduct the study. Written informed consent was sought and obtained from all participants who met the inclusion criteria to protect their right to autonomy. The purpose and objectives of the study were verbally explained to the participants. Participation in the study was voluntary and participants could withdraw from the study at any time. The information gathered from the study was treated confidentially and anonymously. The study was conducted in a private space within the selected primary health care facilities.

## 3. Results

### 3.1. Demographic Characteristic of the Participants

**Figure 1:**
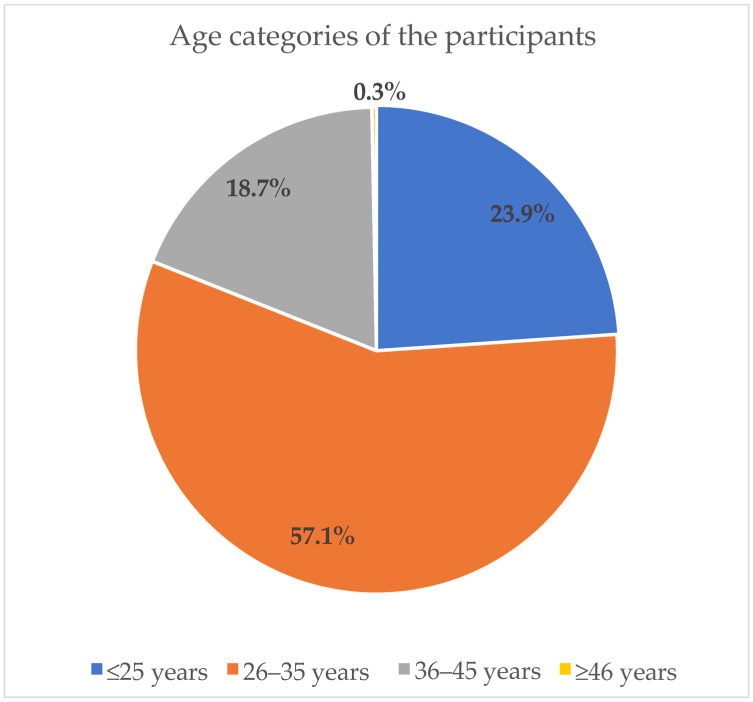
Age categories of the participants in percentages.

Figure 1 shows the age distribution of the participants. The participants were divided into four age categories where the majority (57.1%) of the participants fell within the 26–35 years category. Only a very small (0.3%) percentage was within the 46 years category.

The socio-demographic characteristics of the participants are presented in Table 1. The table shows that almost half of the participants were single (43.9%) with 81.3% having secondary education and only 3.7% of the participants never having attended school. Most of the participants were unemployed (77.9%), and, although 79.8% had a partner who was working, only 15.6% had a household income above $380 per month. More than one third of the participants (38.2%) had two children and slightly above one fifth of the participants (24.2%) had a desire to have children in the future.

A large proportion of the participants practiced Christianity (91.4%) while 24% followed a traditional religion. Being Muslim and practicing no religion were the two lowest categories among the participants at 0.6% each.

**Figure 2:**
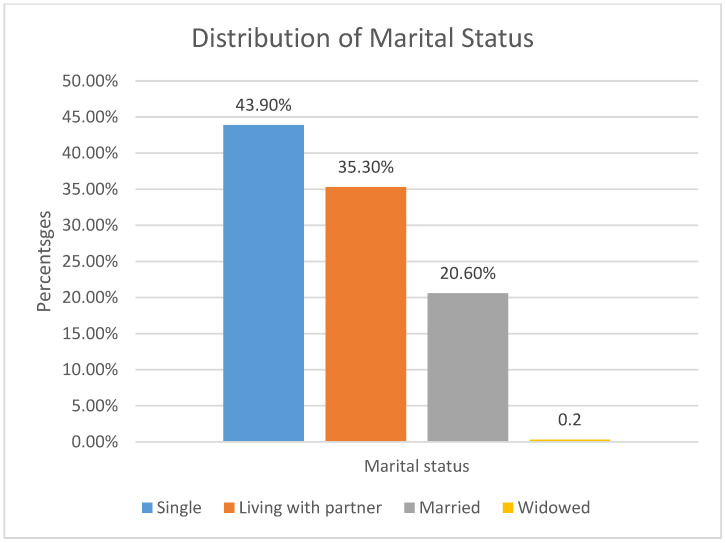
Distribution of marital status.

Figure 2 illustrates the distribution of the marital statuses of the participants in the study. The majority (43.9%) of the participants were single, while living with a partner accounted for 35.3% of the participants; only 20.6% of the participants were married.

Table 2 shows the distribution of the independent variables for sexual behaviour and condom use. Almost half (54%) of the participants had their first HIV diagnosis before pregnancy, with only 0.3% reporting not having received any information about HIV during pregnancy, while the rest (99.7%) of the participants had received it. Most participants (84.4%) were in monogamous relationships. The majority (92.6%) had disclosed their HIV status to their partners, while 72.1% knew their partner’s status. Regular condom use among the participants was reported by 63.2%, while 83.1% of them reported having used condoms during their last sexual encounter. Negotiating for condom use was not a problem among 66.3% of the participants. The majority of the participants had never taken part in risky sexual behaviour within the past year.

**Figure 3:**
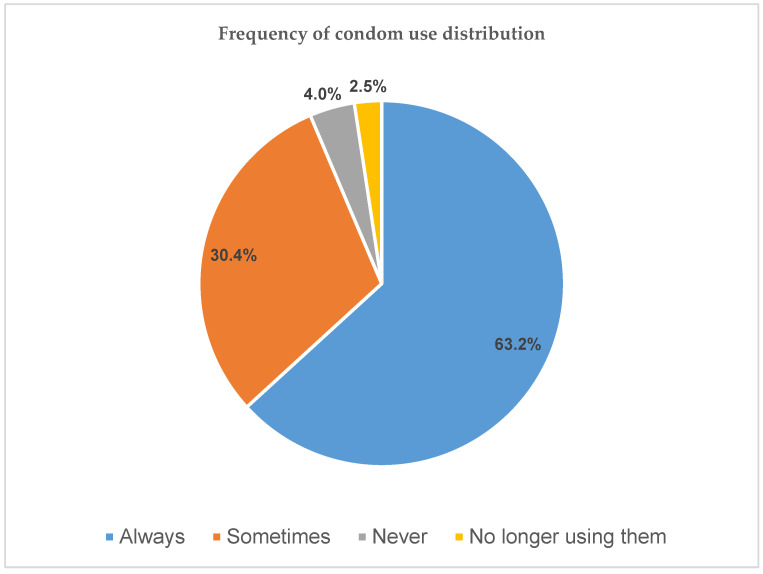
Condom use frequency amongst the participants.

In Figure 3, participants had three options when answering the question “How often do they use condoms during their sexual encounters”, which was used to evaluate regular (consistent) and non-regular (inconsistent) use of condoms amongst the participants. Option 4 which was “no longer using them” emerged as a common answer given by a small percentage (2.5%) of the participants. Reporting “always” was regarded as regular use of condoms, while “sometimes”, “never” and “no longer using them” were regarded as non-regular use of condoms.

**Figure 4:**
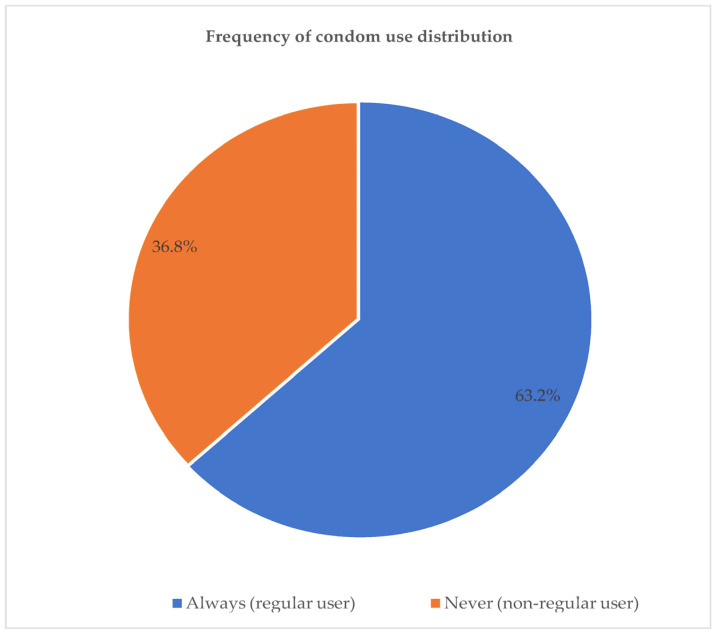
Condom use frequency amongst the participants (merged).

Figure 4 presents the combined percentages of the frequency of condom use uptake during sexual encounters amongst the participants. The participants who reported using condoms sometimes, never, and that they were no longer using them were grouped as non-regular users, whereas those reporting “always using a condom” were classified as regular users. Regular condom use during sexual encounters accounted for 63.2% of the participants.

**Figure 5:**
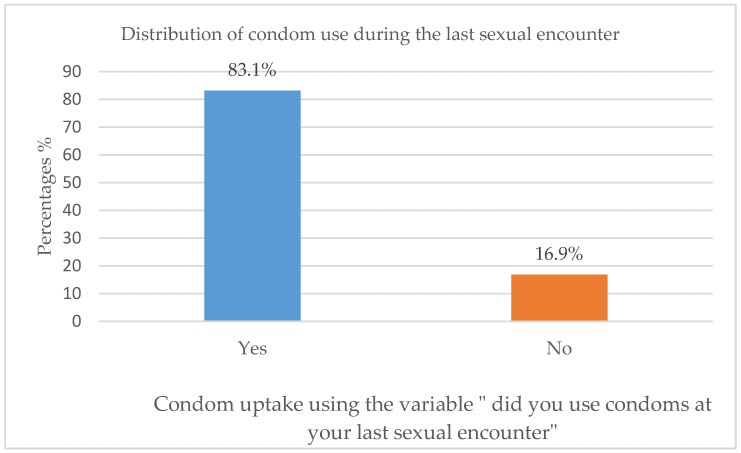
Condoms use at last sexual encounter.

Figure 5 shows the uptake of condom use amongst the participants, whereby 83.1% of the participants used condoms at their last sexual encounter, with only a smaller percentage (16.8%) not using a condom.

### 3.2. Measurement of Association between Dependent and Independent Variables

Table 3 and Table 4 show a bivariate analysis evaluating the association between the two dependent variables, frequency of condom use and condom use at the last sexual encounter, with the independent variables. These variables were also included in the multivariate logistic regression model to determine their association when combined to control for possible confounders. In the bivariate analysis, a significant association was found between the frequency of condom use and marital status (*p* = 0.002), employment status (*p* = 0.015), residence (*p* = 0.000), partner’s employment status (*p* = 0.000), household income (*p* = 0.032), first HIV diagnosis (*p* = 0.032) and disclosure of HIV status to partner (*p* = 0.007). The same dependent variable (frequency of condom use) was found to have significant associations in the multivariate analysis with employment status (*p* = 0.002), residence (*p* = 0.000), number of children (*p* = 0.021), first HIV diagnosis (*p* = 0.020), and disclosure of HIV status to partner (*p* = 0.013).

The odds of frequent use of condoms were higher amongst those who were employed compared to those who were unemployed (OR = 1.632). This was also the case with staying in the township compared to the village (OR = 16.314), increased number of children (OR = 1.396), as well as having disclosed the HIV status to the partner (OR = 3.509). Those who were diagnosed with HIV before pregnancy were found to be less likely to use condoms frequently compared to those diagnosed during pregnancy (OR = 0.555).

A significant association was found by bivariate analysis between condom use at last sexual encounter (second dependent variable) and the partners employment status (*p* = 0.000), disclosure of HIV status to partner (*p* = 0.025), knowledge of partner’s HIV status (*p* = 0.012), and the ability to negotiate condom use (*p* = 0.000). The association between condom use at last sexual encounter with the other independent variables by multivariate analysis was only found between the level of education (*p* = 0.022) and the ability to negotiate condom use (*p* = 0.000).

Those with a lower education level were found to be less likely to have used condoms during the last sexual encounter compared to those with a higher education level (OR = 0.672). This was the same with the ability to negotiate condom use, where those who found it difficult to negotiate condom use were less likely to have used a condom during the past sexual encounter (OR = 0.214).

## 4. Discussion

Correct and consistent condom use is important in the prevention of STIs, including HIV/AIDS. Promotion of condom use remains one of the public health strategies used to curb the spread of HIV among sexually active individuals. This is important because of the high prevalence of HIV amongst the South African general population, whereby approximately 13.7% of the general population were living with HIV in 2021. This figure was equivalent to approximately 8.3 million people living with HIV. Adults aged 15–49 years made up almost 19.5% of the population who were HIV-positive [16].

Woldesenbet et al. [17] reported that 30% and 30.7% of South African pregnant women were HIV-positive in 2019 and 2017, respectively.

This study determined the level of, and factors associated with, condom use among HIV-positive postnatal women in primary health care facilities in Tshwane sub-district 1, Gauteng Province. The results of this study revealed increased condom use amongst the studied population. These results are in contrast to previous studies, which reported low levels of condom use among both HIV sero-concordant and sero-discordant individuals during the postpartum period [3,10]. However, a different study [18] revealed that when HIV-positive women disclose to their partners and receive counselling on family planning during their postnatal period, condom use level became high, which could explain the increase in condom use found in our study. Similar findings were reported by Madiba and Letswalo [7] who discovered that having a better understanding of the risks of infection among HIV-positive women was a predictor of high levels of condom use compared to those women who had less understanding. This might be the case in our study as 99.7% (*n* = 325) reported having received information on HIV during pregnancy. This is also supported by the fact that HIV-positive women obtain information on condom use as part of dual contraception guidance during their interactions with health care providers and peer counselling at their PMTCT clinic visits [19].

### 4.1. Factors Influencing Condom Use

#### 4.1.1. Age

The age of an individual has been found to have a significant association with the use of condoms [20]. However, our study did not show this; no association was found between age and the frequency of condom use in both bivariate and multivariate analyses. Analysis of condom use at last sexual encounter also showed no association with age in both bivariate and multivariate analyses. However, age was found to be 1.6 times more likely to influence condom use at last sexual encounter. Alene [21] states that younger women use condoms consistently for contraceptive purposes compared to older women who are perhaps approaching menopause and no longer interested in contraceptives, reflected in low condom use. In addition to this, Shisana et al. [20] found that older women were more likely to be in stable trusting relationships where they used other alternative contraceptives; hence, the low use of condoms among them. In our study, the majority of the participants were of childbearing age and their use of condoms could be for contraceptive purposes as they were never asked if they were using any other form of contraception.

#### 4.1.2. Marital Status

Individuals who are in less formal relationships have been reported to use condoms more consistently [22] when compared to those in more stable relationships. Hence, condom usage is higher at the beginning of a relationship but declines as the relationship continues [23]. The development of trust in long-term relationships is said to be the main reason condom use drops with the length of the relationship. The results of this study revealed an association between the frequency of condom use and marital status only by bivariate analysis, whereas by adjusted multivariate analysis, no association was found between the two. Analysis of the second variable, condom use at last sexual encounter, also showed no association between condom use at last sexual encounter and marital status in both bivariate and multivariate analyses. These results contrast with other reports, which suggest that relationship type is a predictor of condom use as evidenced by the fact that marriage is associated with expanding the family by having children; therefore, there would be less utilisation of condoms in such a relationship. Unstable relationships are said to be characterised by increase in condom utilization, especially when there is more than one sexual partner involved [24]. However, the lack of association between marital status, frequency of condom usage, and condom usage at last sexual encounter, in this study could be attributed to the fact that only 20.6% of the participants were married. The fact that the majority of the participants had no desire to have children in the future could further explain this lack of association. This was also confirmed by the lack of association between the desire to have children, frequency of condom use, and condom use at last sexual encounter.

On average, the majority of the participants had two children and had no desire to have more children in the future. The most common reasons stated by participants for not wanting more children in the future were related to economic factors. Remarkably, being HIV-positive was only reported by 10.5% of the participants as the reason for not having more children in the future. However, the number of children and the frequency of condom use were significantly associated in multivariate analysis, with the odds showing that the number of children the participants had was associated with a 1.3-times greater likelihood of increased frequency of condom use. These findings are similar to those reported in another study where women with smaller families were found to use condoms more regularly [23] than those with big families with more children. Although most of the participants had only one sexual partner, they were never asked if their relationship was stable or casual. In this regard, no associations were found between the frequency of condom use, condom use at last sexual encounter and number of sexual partners in both bivariate and multivariate analyses.

#### 4.1.3. Level of Education, Socioeconomic Status, and Household Income

Level of education showed no association with the frequency of condom use during sexual encounters among the participants. However, in multivariate logistic regression analysis, a significant statistical association was found between condom use at last sexual encounter and the level of education. These findings are in support of the previous study, which found that educational level was a predictor of condom use in the sense that women with high educational levels were more likely to use condoms consistently compared to those with low educational levels [22]. While having a high level of education increases the chances of improved socioeconomic status, which, in turn, reduces the financial dependency of women on their partner, it also puts them in a better position to negotiate condom use. An association was found between the frequency of condom use during sexual encounters and employment status in both bivariate and multivariate analyses. This is also in line with previous findings that women who were independent economically were more likely to use condoms [22]. Another finding, which confirms socioeconomic status as influencing the use of condoms, is an association found in bivariate analysis between the partner’s employment status, frequency of condom use during sexual encounter, and condom use at last sexual encounter. Interestingly, an association was found in bivariate analysis between household income per month and the frequency of condom usage [22]. Davidoff-Gore [25] found that consistent condom use was low in members of households of low socioeconomic status compared to those of high economic status who were more likely to use condoms, and that condom use at last sexual encounter showed no association with household income.

#### 4.1.4. Ability to Negotiate Condom Use

Ability to negotiate condom use was found to be associated with condom use at last sexual encounter, using both bivariate analysis and multivariate analysis. This is simply attributed to the fact that most of the women have secondary education [26], which is known to play a vital role in the use of condoms. In this study, the majority of women (66.3%) experienced no difficulties in negotiating condom use. The fact that the majority of the participants in this study had secondary education could have contributed to their capability to negotiate for condom use during sexual intercourse.

#### 4.1.5. Place of Residence

Place of residence came out as an important factor that had an association with the frequency of condom use among this population based on a significant association found in both bivariate and multivariate analyses. The odds ratio observed also showed that residence of the participants was 14.3 times more likely to predict frequency of condom use during sexual encounters. This concurs with a previous report, which found that there was lower use of condoms in rural areas compared to urban settings [26]. This difference in condom usage was attributed to the fact that in rural areas the teaching of sexual education is forbidden as it is thought to promote promiscuity. The high use of condoms in urban areas is attributed to the fact that condoms are easily accessible, that there is better exposure to condom use promotion, and economic status is better. However, a Nigerian study also suggested that the difference in sexual practices and behaviours between urban and rural settings was greatly influenced by cultural beliefs [27]. While urban women are more likely to report condom usage than rural women, they are also said to be more likely to have multiple sexual partners and to have had an early sexual debut, resulting in higher condom usage. All the above are indicative that residence is an important factor influencing sexual practices and reproductive health outcomes; hence, it remains important to tailor health education programmes on sexual practices and behaviour specifically to each setting.

#### 4.1.6. Religion

Religion has been found to be one of the factors influencing the use of condoms. However, in this study no significant associations were found between religion and both dependent variables, frequency of condom use and condom use at last sexual encounter, in both bivariate and multivariate analyses. However, the odds ratios indicated that religion was 1.3 times more likely to influence the frequency of condom use and 1.1 times more likely to influence condom use at last sexual encounter. The findings in this study are in contrast with those of previous research where religious background was found to influence sexual practices by discouraging the use of condoms, with sexual practices viewed as being meant to unite couples and encourage childbearing [28]. On the other hand, Mubyazi et al. [29] reported a perceived association between the use of condoms and dishonesty in marital relationships, adultery, and promiscuity associated with religious belief that influenced the development of negative perceptions about the use of condoms. In addition to this, Adekanle et al. [11] reported that in some cultures unprotected sexual intercourse is perceived as a means of strengthening the marriage institution; hence, condom use in this situation is associated with unfaithfulness and unnecessary indulgence by partners.

#### 4.1.7. Disclosure of HIV Status

Disclosure of HIV status and frequency of condom use during sexual encounters showed a significant association in multivariate analysis, with the odds ratio showing that disclosure of a woman’s HIV status was 3.5-times more likely to influence the frequency of condom use. When using the second dependent variable, condom use at last sexual encounter, an association was only found in bivariate analysis. However, the odds ratio showed that disclosure of HIV status to partners was 1.4-times more likely to influence condom use at last sexual encounter. These results are consistent with previous findings [7] that suggest that knowing the HIV status of a partner is associated with consistent or regular use of condoms. In addition, disclosure of HIV status has been identified as one of the factors that enables women to negotiate condom use with their partners [8]. Previous reports suggest that members of married couples have a high probability of disclosing their HIV status to their partners as opposed to single persons [9,11]. However, that was not the case in our study as only 20.6% of the participants were married but 92.6% had disclosed their HIV status. Knowledge of a partner’s HIV status was found to have no association with the frequency of condom use. A significant association between knowledge of partner’s HIV status and condom use at last sexual encounter was only found in bivariate analysis. Knowledge of a partner’s HIV status was found to be associated with a 1.4-times increased likelihood of condom use at last sexual encounter.

#### 4.1.8. Risky Sexual Behaviours

Practicing risky sexual behaviours was uncommon in this study, as the majority of the participants had never in the past year had sexual intercourse with a non-regular sexual partner or had sexual intercourse under the influence of alcohol or had group sex. A very small portion of the participants (4%) agreed to have multiple sexual partners in the past year. A previous research report found that condom use was adversely affected by the use of alcohol and other drugs as such use increased the likelihood of engaging in risky sexual behaviours [30].

### 4.2. Limitations of the Study

The major limitation of this study is the fact that convenience sampling was used and only those women who matched the inclusion criteria were included according to their availability. This limits generalization of the results to the entire population. This study included only women; hence, it does not allow evaluation of trends that may differ by gender. All the postnatal women who were included in the study were already on a PMTCT programme that included within it the promotion of consistent condom use; hence, this could have influenced the participant’s responses. The self-reported measures that were used to collect the data and the reporting bias associated with them may have influenced the accuracy of the responses. However, no leading questions were asked, and all the questions were clear, easy to understand and needed short answers only. The sensitive nature of sexuality and HIV infection can lead to some participants withholding more useful information about their sexual experiences and give socially desirable responses. This has been found to be common in many sexual behaviour studies, especially among women and girls. Nevertheless, privacy was maintained in this study throughout the data collection period. Data collection was conducted in a private room and participants were informed that all the information shared was confidential and that they would remain anonymous.

## 5. Conclusions

This study revealed improved condom uptake amongst HIV-positive postnatal women, which was evidenced by more than half of the participants reporting that they always used condoms during sexual encounters. The use of condoms in this study was found to be greatly influenced by factors such as level of education, employment status, residence, number of children, first HIV diagnosis, disclosure of HIV status to partner, and the ability to negotiate condom use. While these findings indicate major improvements in condom use, especially in this population deemed to be vulnerable, further research is needed specifically among this population to further understand the factors influencing sexual behaviours.

## 6. Recommendations

- HIV prevention strategies should be directed to HIV-positive individuals as a means of prevention including of transmission of resistant strains of HIV, and as a means of protecting their partners in sero-discordant relationships.
- This study shows that programmes aimed at improving educational level among HIV postnatal women could be used as a strategy to further increase condom use amongst this group.
- It remains vital that health education messages aimed at promoting condom use amongst HIV-positive postnatal women are personalised to each setting and target barriers identified in this study.
- Partner involvement during promotion of the use of condoms is needed in this population to facilitate easy negotiation and shared decision making on condom use.
- It is also recommended that further research on barriers to condom use amongst HIV-positive women be undertaken to obtain a holistic understanding of the barriers that have been identified associated with condom use in this study.

## Figures and Tables

**Table 1:** Participant’s socio-demographic information (*n* = 326).

Variables	Responses	Frequency	Percentages
Marital status	Single	143	43.9
	Living with a partner	115	35.3
	Married	67	20.6
	Widowed	1	0.3
Education level	Primary school	22	6.8
	Secondary	265	81.3
	College	18	5.5
	University	9	2.8
	Never attended school	12	3.7
Employment	Employed	60	18.4
	Self employed	12	3.7
	Unemployed	254	77.9
Is partner working?	Yes	260	79.8
	No	50	15.3
	I have no partner	16	4.9
Household income per month	$0–$152	142	43.6
	$160–$380	133	40.8
	Above $380	51	15.6
Number of children	1	65	19.9
	2	124	38.2
	3	78	23.9
	4	42	12.9
	5	14	4.3
	6	2	0.6
	7	1	0.3
Desire to have children	Yes	79	24.2
	No	247	75.8
Religion	Christian	298	91.4
	Traditional religion	24	7.4
	Muslim	2	0.6
	None	2	0.6
Residence	Township	I57	48.2
	Village	164	50.3
	Informal settlement	5	7.5

**Table 2:** Distribution of independent variables for sexual behaviour and condom use.

Variable	Responses	Frequency	Percentages
First HIV diagnosis	Before pregnancy	176	54
	During pregnancy	146	44.8
	After pregnancy	4	1.2
Received information on HIV during pregnancy	Yes	325	99.7
	No	1	0.3
Number of sexual partners in the past two years	1	275	84.4
	2	43	13.2
	3	5	1.5
	4	2	0.6
	6	1	0.3
Disclosed HIV status to partner	Yes	302	9 2.6
	No	24	7.4
Knowing partner’s HIV status	Yes	235	72.1
	No	91	27.9
How often do you use condoms during sexual encounter?	Always	206	63.2
	Sometimes	99	30.4
	Never	13	4
	No longer using them	8	2.5
Used condoms at last sexual encounter	Yes	271	83.1
	No	55	16.9
Difficulty in negotiating condom use	Yes	106	32.5
	No	216	66.3
	Never negotiated	4	1.2
Risky sexual behaviour in the past year	Multiple sexual partners	13	4
	Group sex	1	0.3
	Sexual encounter under the influence of alcohol	1	0.3
	Sexual encounter with a non-regular partner	2	0.6
	No risky sexual behaviour	309	94.8

**Table 3:** Association between the dependent variable “How often do you use condoms” and various independent variables.

Dependent Variable “How Often Do You Use Condoms” Reported as “Regular Use and Non-Regular Use”				*p*-Value at Multivariate Analysis
Independent Variables	Odds Ratio	95% Confidence Interval	*p*-Value at Bivariate Analysis	
Age	0.673	0.784–1.051	0.133	0.083
Marital status	0.940	0.123–0.226	0.002	0.715
Education level	1.029	0.056–0.113	0.099	0.872
Employment status	1.632	0.178–0.799	0.015	0.002
Religion	1.326	0.392–0.631	0.691	0.410
Residence	14.314	0.258–3.086	0.000	0.000
Number of children	1.396	0.049–0.611	0.238	0.021
Desire for children in future	1.124	0.521–1.278	0.805	0.717
Partner’s employment status	0.784	0.465–0.812	0.000	0.432
Household income per month	1.070	0.280–0.700	0.032	0.695
First HIV diagnosis	0.555	0.091–1.0780	0.032	0.020
Disclosure of HIV status to partner	3.509	0.266–2.244	0.007	0.013
Knowledge of partner’s HIV status	0.985	0.266–2.406	0.096	0.955
Number of sexual partners in the past 2 years	0.926	0.537–0.920	0.154	0.744
Ability to negotiate condom use	0.966	0.547–0.612	0.374	0.902

**Table 4:** Association between the dependent variable, “did you use condoms the last time you had a sexual encounter”, and the independent variable, “did you use condoms the last time you had a sexual encounter”, reported as “yes or no”.

Independent Variables	Odds Ratio	95% Confidence Interval	*p*-Value at Bivariate Analysis	*p*-Value at Multivariate Analysis
Age	1.614	0.735–1.535	0.767	0.150
Marital status	1.334	0.165–0.734	0.052	0.215
Education level	0.672	0.064–0.870	0.325	0.022
Employment status	0.981	0.494–0.865	0.566	0.780
Religion	1.148	0.802–2.001	0.349	0.698
Residence	1.458	0.304–1.059	0.975	0.278
Number of children	0.773	0.730–1.260	0.734	0.246
Desire for children in future	1.077	0.458–1.508	0.647	0.585
Partner’s employment status	0.759	0.941–1.341	0.000	0.062
Household income per month	1.243	0.133–0.844	0.065	0.188
First HIV diagnosis	1.596	0.152–1.150	0.136	0.140
Disclosure of HIV status to partner	1.417	0.465–1.968	0.025	0.369
Knowledge of partner’s HIV status	1.442	0.108–1.536	0.012	0.096
Number of sexual partners in the past 2 years	0.809	0.730–1.155	0.503	0.605
Ability to negotiate condom use	0.214	0.565–2.945	0.000	0.000

## Data Availability

The data presented in this study are available on reasonable request from the corresponding author. The data are not publicly available due to ethical requirements.
